# Money talks: performance-based reimbursement systems impact on perceived work, health and patient care for physicians in Sweden

**DOI:** 10.3389/fpsyg.2023.1216229

**Published:** 2023-07-07

**Authors:** Emma Brulin, Kerstin Ekberg, Bodil J. Landstad, Ulrik Lidwall, Malin Sjöström, Alexander Wilczek

**Affiliations:** ^1^Unit of Occupational Medicine, Institute of Environmental Medicine, Karolinska Institutet, Stockholm, Sweden; ^2^Department of Health, Medicine, and Caring Sciences (HMV), Linköping University, Linköping, Sweden; ^3^Faculty of Human Sciences, Mid Sweden University, Östersund, Sweden; ^4^Unit of Research, Education and Development, Östersund Hospital, Östersund, Sweden; ^5^Division of Insurance Medicine, Department of Clinical Neuroscience, Karolinska Institutet, Stockholm, Sweden; ^6^Official Statistics Unit, Department for Analysis, Swedish Social Insurance Agency, Stockholm, Sweden; ^7^Department of Public Health and Clinical Medicine, Umeå University, Umeå, Sweden; ^8^Department of Clinical Sciences, Danderyd Hospital, Karolinska Institutet, Stockholm, Sweden

**Keywords:** mixed method, performance-based reimbursement, physicians, patient care, work environment, stress-induced exhaustion disorder

## Abstract

**Introduction:**

The study aimed to investigate in which way performance-based reimbursement (PBR) systems in Swedish healthcare services (1) subjectively impacted physicians’ work and patient care and (2) were associated with the occurrence of stress-induced exhaustion disorders among physicians.

**Method:**

The study applied a mixed-method design. Data were collected from a representative sample of Swedish physicians. In the questionnaire, respondents were asked to answer an open-ended question regarding their reflections on PBR. The answers to the open-ended question were analysed using thematic analysis. Respondents were also asked to rate the impact of PBR on their work. The association between PBR and self-rated stress-induced exhaustion disease was analysed with logistic regressions. Stress-induced exhaustion disorder was measured using the Burnout Assessment Scale.

**Results:**

Thematic analysis resulted in four themes: (1) Money talks, (2) Patients are affected, (3) Medical morals are challenged, and (4) PBR increase the quantity of illegitimate tasks. Logistic regressions showed that physicians who experienced PBR had an impact on their work and had a two-fold higher risk of stress-induced exhaustion disorder.

**Discussion:**

Our findings suggest that current reimbursement systems in Sweden play an essential role in Swedish healthcare and negatively influence physicians’ work and health. Also, current PBR impact patients negatively. No previous study has explored the potentially harmful impact of PBR on how physicians perceive work, health and patient care. Results indicate that policymakers should be encouraged to deeply review PBR systems and focus on ways that they can limit the negative impact on physicians’ work and health while meeting future challenges.

## 1. Introduction

In early 1990, New Public Management (NPM) was introduced as a management method in public organisations (Agerberg, 2014; Funck and Karlsson, 2020). The purpose of NPM was to increase internal and external efficiency, i.e., cut costs, improve productivity, and increase patient satisfaction (Funck and Karlsson, 2020). Performance measurement, quantifying care provision, and the economisation of healthcare are commonly associated with NPM (Johansson and Siverbo, 2009). In Sweden, NPM resulted in that inpatient and outpatient clinics at hospitals were, and still are, governed as production units with defined production goals. Clinics in the hospital and primary healthcare facilities have unit-level financial incentives through performance-based reimbursements (PBR) systems (Quaye, 2001; Vengberg et al., 2021). A recent study in Swedish primary healthcare showed that regardless of the kind of payment, primary healthcare physicians experienced PBR impacted care provision (Vengberg et al., 2021). They expressed that PBR stimulates the production of shorter patient consultations, up-coding of consultations and skimming for healthier patients (Vengberg et al., 2021). As such, PBR becomes manifested in professionals’ work and transforms contexts in which medicine is practised (Fırtın and Karlsson, 2020; Vengberg et al., 2021). In this study, we will make use of the Healthy Healthcare concept (De Lange et al., 2020; Løvseth and de Lange, 2020) and a mixed method design, to investigate how PBR systems in Swedish healthcare (1) subjectively impacted physicians’ work and patient care, and (2) were associated with the occurrence of stress-induced exhaustion disorder.

### 1.1. Linking healthcare organisation, physicians’ occupational health and patient care

The Healthy Healthcare concept acknowledges the link between the organisation, healthcare professionals and quality of care (Teoh et al., 2019). It is intended to convey the importance of good health among healthcare professionals as a competitive advantage in organisations striving to deliver resource-efficient, high-quality care to patients (De Lange et al., 2020; Løvseth and de Lange, 2020). The Healthy Healthcare concept emphasises the complex and interdependent relationships between three healthcare service dimensions known to impact healthcare, referred to as the three pillars (De Lange et al., 2020; Løvseth and de Lange, 2020): (i) organisational practises, (ii) healthcare professionals’ occupational health and wellbeing, and (iii) quality of care. The Healthy Halthcare concept guides us in situating the individual physicians in a dynamic context consisting of colleagues, organisational and professional cultures, structures, politics and financial systems. In the following this context is further described.

At large, the NPM reforms included deregulation, privatisation and marketisation at a local level. The reforms were associated with contractor-models, standardisation, quantification and measurement of performances resulting in performance-based reimbursement systems (Knutsson et al., 2017; Lapsley, 2017). Although the reforms have been shown to have both positive and negative effects, the criticism of the reforms has been extensive. For example, the extensive use of performance measurement has been criticised because of its unintended consequences and concerns have been raised that the implementation of PBR systems stimulates shorter visits, up-coding of visits and skimming of healthier patients (Vengberg et al., 2021; Höglund et al., 2023) and thereby have displacement effects on care-incentives and socio-economically vulnerable patient groups (Anell et al., 2012).

The managerial changes in NPM could thus pose a situation where professional tasks trigger a conflict between the physicians’ values and the clinic’s routines and goals. This could cause moral distress (Førde and Aasland, 2008) and become hazardous to physicians’ health (Maslach and Jackson, 1981). In 2015, the Swedish Medical Association conducted a survey of a large share of general practitioners (GPs) in Sweden. The survey included questions about the clinic’s reimbursement and financial system. It turned out that only 19% of GPs in Sweden had complete confidence in their clinic’s “system for contracts and reimbursements.” A vast majority, 79% of the GPs fully or partially agreed with the statement: “The reimbursement system means that I cannot work according to the ethical principles I wish to follow” (unpublished data). These findings are supported by the study of Vengberg et al. (2021).

Since the introduction of NPM, the possibility for Swedish physicians to exercise occupational control and their autonomy in clinical work has changed (Bejerot et al., 2011, 2017; Aronsson et al., 2012). The amount of working time spent on core tasks has decreased, administrative tasks have increased, and everyday physicians experience too many illegitimate tasks (Aronsson et al., 2012). NPM and the introduction of PBRs have contributed to reduced occupational control and change in clinical work, as well as to higher demands and lower individual control and support among physicians in Sweden (Bejerot et al., 2011, 2017). In 2010 compared to early 1990, when NPM was first introduced, physicians in Sweden experienced less support from the organisation, a greater distance to the closest manager and less control both of their own work as well as of the organisation of their work (Bejerot et al., 2011). Also, organisational changes like the introduction of Lean management resulted in physicians experiencing a loss of support from colleagues and a deterioration of control (Bejerot et al., 2017). It is well established that when demands increase and job control decreases, as described in the studies above, the risk of stress-induced exhaustion disorder increases (Häusser et al., 2010; Theorell et al., 2015; Harvey et al., 2017). Furthermore, low workplace support, organisational change, and atypical working hours are important contributors to individuals’ developing stress-induced exhaustion disorders (Harvey et al., 2017).

Healthcare services in Sweden are organised under 21 self-governing regional authorities, called Regions, responsible for providing a significant proportion of all public healthcare services in hospitals and primary healthcare facilities (Mattson and Peterson, 2003). The Regions are governed by political assemblies that have a considerable degree of autonomy and make decisions on budget and how payments within PBR should be distributed. Payments to healthcare providers in Sweden may differ between healthcare regions, but they are all performance-based to some extent (Vengberg et al., 2021). Although PBR in Swedish healthcare at large entails that the more consultations, measures, interventions, and treatment procedures are carried out, the more money for the clinic, there are differences between the regions.

Interview data collected in a psychiatric clinic for healthcare professionals on sick leave due to stress-induced exhaustion disorder identified two factors which, in addition to a heavy workload, had contributed to their stress-induced exhaustion disorder (data not published). These were: *lack of control* and *ethical stress*. These factors are in line with previous studies (Bejerot et al., 2017; Vengberg et al., 2021). Based on these findings, questions about PBR were included in a large data collection among a representative sample of physicians working in Sweden in 2021 (Hagqvist et al., 2022). In the present study, we intended to analyse this data to gain more knowledge about PBR in relation to physicians’ work, health and perceived patient care. To the best of our knowledge, no previous study has used an Healthy Healthcare perspective to explored the role of PBR systems in relation to (i) occupational health and wellbeing, and (ii) perceived quality of patient care.

## 2. Study design and method

This study used an embedded mixed-method design involving the integration of qualitative findings and quantitative results (Pluye and Hong, 2014). In this study, the results from the qualitative analysis were used to design the quantitative analysis.

Both the qualitative and the quantitative material derives from data in the Longitudinal Occupational Health survey in Healthcare Sweden (LOHHCS). LOHHCS data was collected from a representative sample of practising physicians in Sweden from February to May 2021 (Hagqvist et al., 2022). Using a stratified random sampling method, a total of 6,699 physicians were drawn from the Swedish Occupational Register held by Statistics Sweden. The response rate was 41%. The cohort included 44.8% male and 55.2% female physicians. The mean age of the cohort was 47.5 years, ranging from 27 to 77 years. To adjust for missing data and stratifications, Statistics Sweden calculated calibrating weights which are applied in this study giving us an analytical sample of 33,703. Study design is described in detail in Hagqvist et al. (2022). The Swedish Ethical Review Authority approved this study (2020-06613).

The LOHHCS questionnaire involved 79 questions and scales on physicians’ work and health (Hagqvist et al., 2022). In addition, physicians were asked to report their place of work (i.e., in hospital or primary healthcare facilities) and hierarchical position.

### 2.1. Qualitative material

In the LOHHCS questionnaire, physicians were encouraged to respond to an open-ended question about their experience of how PBR impacted them. A total of 334 physicians wrote about their experiences. One hundred thirty responses (39%) were not relevant in relation to the purpose of this study and were removed, resulting in 204 remaining responses. The open-ended responses were analysed by the first author using a thematic analysis (Braun and Clarke, 2006). Analysis was conducted in four steps. First, a large number of responses were read, and six empirical themes emerged. Second, all responses were read through several times and coded based on the six themes that emerged in the first step. Third, when all responses were coded, four of the original themes were merged into two separate themes. The final analysis of the material thus resulted in a total of four themes (Table 1). Fourth, as in the example in Table 1, all codes were given a line in a spreadsheet. The number of codes in each of the four themes was summarised. Some responses were rich and included aspects from different themes. These were then coded to all matching themes (see example in Table 1).

**Table 1:** Examples of response and how they were coded to themes marked by an X.

	Themes			
Responses that were coded to themes.	Money talks	PBR increases the quantity of illegitimate tasks	Medical morals are challenged	The patients are affected
Taking many quick visits is valued more than fewer and longer ones. This disadvantages elderly and multi-morbid patients, which contributes to high ethical stress at work.	X		X	
Setting a diagnosis to every little note in the medical record takes time and makes the medical record unmanageable, resulting in less patient time and reduced safety.		X		X
Extra administration.		X		

### 2.2. Quantitative material

#### 2.2.1. Measurements

*Performance-based reimbursement (PBR)* systems were measured using three questions from the LOHHCS questionnaire. First, physicians were asked to what extent they experienced PBR to impact their work, with answers on a 4-point scale ranging from “to a large extent” to “not at all.” Answers were dichotomised into PBR impact work (1) using the two first options, and PBR does not impact work (0).

Second, those who answered that PBR impacted their work were then asked to rate, on a 4-point scale, whether the experience of how PBR impacted their work was “very positive,” “positive,” “negative,” or “very negative.” The variable was dichotomised into positive and negative.

Third, physicians were asked to what extent they experienced that PBR affected their ability to act on patients’ medical needs. Answers ranged on a 4-point scale: “to a very large extent,” “to a large extent,” to “to some extent,” and “not at all.” Answers were dichotomised into PBR impact care provision using the two first options, and PBR does not impact care provision.

Based on the results of the qualitative analysis we included an additional four variables in the quantitative analysis. These four variables were: *Perceived autonomy in patient care*, *illegitimate tasks*, *moral distress*, and *work site*.

Perceived autonomy in patient care was measured using three questions. Physicians were asked to what degree they felt that they had (1) the time needed in patient consultations, (2) the freedom to make clinical decisions meeting the patient’s needs, and (3) the possibility to provide all patients with high-quality care. Answers on a 5-point scale ranged from a large degree to a small degree. A grand mean for the three variables was computed, ranging from 1 high autonomy to 5 low autonomy (Cronbach’s alpha = 0.692).

To measure illegitimate tasks, the Bern Illegitimate Task Scale (BITS) was used (Jacobshagen, 2006; Semmer et al., 2010). BITS asks the respondents how often they conduct unnecessary tasks and unreasonable tasks, with answers ranging from never (0) to frequently (4). A grand mean was computed based on the eight items. In total scale, a higher number indicates a high frequency of illegitimate tasks. The scale had a high internal consistency (Cronbach alpha’s = 0.862).

Moral distress comprised 10 items asking the respondents to rate how stressful they experience certain situations. Examples of situations are “that patients receive poor care due to economic reasons” or “when I now and then need to act against my conscious.” Cronbach’s alpha for moral distress was 0.853. A grand mean was computed based on the 10 items.

Perceived autonomy in patient care, illegitimate tasks, and moral distress were dichotomised based on the upper quartile.

The work site was coded into primary or hospital healthcare facilities.

Last, the variable self-rated *stress-induced exhaustion disorder* was measured using the validated Burnout Assessment Tool (BAT) (Schaufeli et al., 2020b). The BAT includes 23 items with answers on a 5-point scale from not at all to almost all the time. All items were added to a grand mean (Cronbach’s alpha = 0.862), and a cut-off value was set at 2.59. According to guidelines by Schaufeli et al. (2020a,b), a mean BAT-score of 2.59 and above indicates a high risk of developing clinical burnout or that the person already has a sever burnout, i.e., stress-induced exhaustion disease. Stress-induced exhaustion disorder was used as the outcome measures in the statistical analysis.

#### 2.2.2. Analysis

The quantitative analyses were carried out in four steps. First, descriptive statistics were computed to show distributions across the included variables for the total sample and for primary and hospital care physicians, respectively. Potential differences between primary and hospital care physicians were assessed using chi-square tests.

Second, the levels of perceived autonomy in patient care, illegitimate tasks, moral distress, and stress-induced exhaustion disorder between physicians who experienced an impact from PBR and those who did not were explored using independent *t*-tests. *T*-tests were also computed stratified for primary and hospital care physicians.

Third, to assess the association between PBR and stress-induced exhaustion disorder, logistic regressions were conducted for the calculation of odds ratios (OR) with 95% confidence intervals (CI). First, crude unadjusted values for all included variables were computed in relation to stress-induced exhaustion disorder. In Models 1–3, the three PBR variables were explored respectively, adjusted for perceived autonomy in patient care, illegitimate tasks, moral distress, work site, and rank in relation to stress-induced exhaustion disorder.

Last, the logistic regressions were computed stratified for primary and hospital care physicians.

## 3. Results

### 3.1. The experienced effect of PBR on physicians’ work

The thematic analysis resulted in four themes: (1) money talks; (2) the patients are affected; (3) medical morals are challenged; and (4) PBR increases the quantity of illegitimate tasks.

Physicians working in primary healthcare facilities responded to a greater extent to the open-ended question used in the thematic analysis than physicians working in hospitals (148 and 56 responses, respectively).

Of the original 334 responses, only three wrote that they had positive experiences regarding the PBR systems. Because of the few positive responses not reaching saturation, they are not included in the 204 responses used in the analysis.

#### 3.1.1. Money talks

In this theme, physicians describe that healthcare is governed by money rather than patients’ needs. Thirty-six responses were coded to the theme Money talks, 24 for physicians in primary healthcare and 12 for physicians in hospitals.

In their responses, physicians wrote that care was availability-driven and not need-driven. Physicians were prompted to take on patients that could result in higher income for the care facility.

*“Complicated patients are booked for shorter consultations times than desired because we have to see more patients for “sticks” to be counted.” (Author’s note sticks refer to a line in the statistics).*

Physicians described that their work is adapted to performance incentives linked to specific patients, consultations, or treatments. For example, because a general health assessment gives extra reimbursement, these are prioritised in primary healthcare, regardless of indication. Primary healthcare physicians described that if a patient has several medical conditions, he or she is booked for one consultation per medical condition rather than handling all conditions at one consultation. Also, in the emergency unit, incentives are linked to reduced patient waiting time. This results in care being steered towards taking on waiting patients with mild medical conditions rather than those who come to the emergency unit with more critical or time-consuming medical conditions.

*“The work is adapted to what the manager believes gives the best compensation, not what is medically relevant.”*

Clinics are often paid more for physical patient consultations with physicians than with nurses. This results in physicians being booked for patient consultations that could instead be handled by nurses directly.

*“Patients with less serious illnesses are prioritised as they provide a good income. Even those patients that do not need a medical assessment or suffice with simple advice or, at most, a visit to a nurse [are booked to physicians’ consultations]. This can lead to irritation/moral stress about how tax money or healthcare resources are used, and sometimes it also leads to lack of time/stress for the more serious cases.”*

#### 3.1.2. The patients are affected

The theme describes how physicians experienced that the PBR steer care provision towards less complicated and more healthy patients, pushing out those in real need of care. The theme also describes how patients, in the end, are affected by the PBR system. A total of 58 responses were coded into this theme, 46 made by physicians in primary healthcare and 12 made by physicians in hospitals.

The physicians experienced that patients in need of extensive care are disadvantaged in the PBR system. They expressed that they have no or very little time for older patients or patients with multiple diagnoses.

*“Taking many quick visits is valued more than fewer and longer ones. This is a disadvantage for elderly and multi-ill patients, which contributes to high ethical stress at work.”*

Physicians expressed that they do not have the time they need to follow up on patients. When they follow up on patients, especially chronically ill patients, it must be adjusted to the PBR and not to the patient’s needs. For instance, taking blood samples of diabetic patients could easily be handled by sending the patient directly to the lab, and then the physicians read the test results and adjust medications. However, for the clinic to be reimbursed for the work, the patient also needs a physical consultation with a physician. Another example expressed by the physicians is that the PBR does not fully reimburse phone calls which are often used to follow up on older and multi-ill patients. This can harm the older and make continuity of care more difficult.

*“Handling medicines, follow-up of samples, etc. for elderly people with multiple illnesses is often done by phone, partly because they have difficulty getting to the primary health centre, partly to protect them from possible infection. This is often done on non-existent time and, as far as I know, provides little compensation.”*

Primary healthcare physicians described that preventive and promotive care were not prioritised. They expressed that care becomes short-term and that long-term care that aims to reduce hospitalisation is neglected.

#### 3.1.3. Medical morals are challenged

The theme describes how physicians experienced PBR challenges in medical practices and the moral stress inflicted on them. The hunt for reimbursement impacted their ability to practice medicine and resulted in moral stress among physicians. Performance and production became more central than the patients themselves. Sixty-seven responses were coded in the theme of Medical morals are challenged. Primary healthcare physicians contributed 45 codes to this theme, and physicians working in hospitals 22 codes.

Physicians described how they experience that PBR steered their work in such a direction that it affected the quality of care negatively.

*“Due to the requirements of production, productivity and how many minutes it takes to handle a patient are often measured, while qualitative parameters of care given, or patient satisfaction is not measured.”*

Physicians express that the focus on production rather than care is stressful and against medical practices.

*“It affects my ability to, based on competence and judgement, create the healthcare that is needed to meet the needs of those who need healthcare resources the most.”*

Physicians also described feeling stressed about fitting patients’ consultations to predetermined timeslots. They experienced high demands on productivity, i.e., seeing many patients each day. They should manage a certain number of consultations each day. Time slots do not take patients’ needs into consideration.

*“All the focus is on seeing as many patients as possible. The fact that the patient is 95 years old, bedridden or needs an interpreter is not taken into account when appointments are booked. It causes stress because it is not possible to keep time.”*

Care is also provided by administrating new prescriptions or by phone consultations. This type of care provision gives little or no financial reimbursement. Physicians described how they struggle to find the time for this type of care provision.

*“Quantity is reimbursed, not quality. The number of consultations is all that counts, not what you actually do. We get reimbursed more to look at a birthmark or tick bite for 1 min at a physical patient meeting than to, for example, have a 30-min productive phone call with a depressed patient or do a review of the medical record, or write a detailed referral to help a patient further, etc., such is not compensated at all.”*

#### 3.1.4. PBR increases quantity of illegitimate tasks

The theme describes how physicians experience that PBR increases administration and that these tasks often are unnecessary and unreasonable. Illegitimate tasks received 87 codes, of which 69 came from primary healthcare physicians and 18 from physicians working in hospitals.

The PBR presuppose that physicians register patient meetings and their diagnoses. For each patient, specific diagnoses or action codes need to be registered in a data system. Physicians described that this registration of patients’ diagnoses or action codes is unnecessary for physicians to carry out.

*“Register diagnosis codes solely to ask for more money feels like an unnecessary task (for physicians).”*

Physicians expressed that the time used on administrative work is time that could be used on direct work with patients instead.

*“I estimate that 20% of my work time is used on administrative tasks related to the PBR.”*

Physicians also described how they need to use their free time to have time for administration. They are often not compensated for the after-work hours they put in.

*“I usually do not have enough time to administration and need to stay longer at the hospital to catch up with admin. However, there is no flexitime, and I am not compensated for overtime. If I do not stay long, administration that affects patients’ medical needs is waiting to make timely decisions.”*

### 3.2. Descriptives and the association between PBR and risk of stress-induced exhaustion disorders

Starting with descriptive data, Table 2 shows that 12.9% of physicians in Sweden had a risk of developing or already had developed stress-induced exhaustion disorder in the spring of 2021, with a higher prevalence among physicians working at hospitals, than in primary healthcare facilities. Every fifth physician in Sweden reported that PBR impacts their work (20.5%). Among those who reported that PBR affect their work, 77.2% experienced a negative impact on their work. Compared to hospital-based physicians, a slightly larger share of primary healthcare physicians responded that they experienced a negative impact from PBR. 25.7% of all physicians experienced that PBR impacted care provision.

**Table 2:** Descriptive statistics with chi-square for differences between categories across the included variables.

	Total	Primary healthcare	Hospital	Sig.
*N*	33,703	8,220 (24.4%)	25,483 (75.6%)	
Stress-induced exhaustion disease	12.9%	11.8%	13.3%	0.002
PBR^a^ impact work	20.5%	35.6%	14.9%	<0.001
PBR^a^ impact work negatively	77.3%	87.6%	71.2%	<0.001
PBR^a^ impact care provision	25.7%	32.4%	21.9%	<0.001
Autonomy in patient care^b^:				
Low autonomy	31.7%	39.4%	28.6%	<0.001
High autonomy	68.3%	60.6%	71.4%	
Moral distress^b^:				
High level of moral distress	26.8%	28.2%	26.1%	0.030
Low levels of moral distress	73.2%	71.8%	73.9%	
Illegitimate tasks^b^:				
Frequently	26.7%	29.7%	25.6%	<0.001
Seldom	73.3%	70.3%	74.4%	

a Performance-based reimbursement scheme.

b Dichotomised based on the upper quartile.

Overall, 31.7% of practising physicians in Sweden rated that they have low autonomy in patient care. Almost 27% reported high levels of moral distress and frequent illegitimate tasks, respectively. Compared to hospital-based physicians, significantly more physicians in primary healthcare rated their autonomy in patient care as low, with high levels of moral distress and frequent illegitimate tasks.

Comparisons of mean levels of autonomy in patient care, moral distress, and frequency of illegitimate tasks showed significant differences between physicians who experienced that PBR impact their work, compared to those who did not experience that PBR impact their work (Table 3). Across all PBR variables, those who rated impact of PBR scored significantly higher on autonomy in patient care, moral distress, and frequency of illegitimate tasks. Also, the mean level of stress-induced exhaustion disorder is higher among physicians who experience PBR impacted them.

**Table 3:** T-test with the mean, standard deviation for autonomy in patient care, moral distress, frequency of illegitimate, and stress-induced exhaustion disorder between physicians who experience an impact of performance-based reimbursement (PBR) compared to those who do not.

	Autonomy in patient care (scale 1–5)^b^	Moral distress (scale 1–4)^b^	Frequency of illegitimate tasks (scale 0–4)^b^	Stress-induced exhaustion disorder (scale 0–5)^b^
PBR^a^ impact work	2.57 (0.80)**	3.10 (0.57)**	2.31 (0.77)**	2.04 (0.64)**
PBR^a^ does not impact work	2.18 (0.66)**	2.85 (0.60)**	1.92 (0.71)**	1.81 (0.59)**
PBR^a^ impact work negatively	2.52 (0.72)**	3.06 (0.54)**	2.29 (0.74)**	2.02 (0.63)**
PBR^a^ impact work positively	2.03 (0.66)**	2.77 (0.68)**	1.78 (0.72)**	1.71 (0.71)**
PBR^a^ impact care provision	2.63 (0.90)**	3.24 (0.51)**	2.38 (0.84)**	2.10 (0.73)**
PBR^a^ does not impact care provision	2.33 (0.65)**	2.90 (0.58)**	2.10 (0.72)**	1.90 (0.58)**

**p < 0.001 for between groups.

a Performance-based reimbursement.

b A higher score indicates a worse situation/experience.

In Table 4, the results from the logistic regressions are presented. The unadjusted crude value shows that a negative experience of PBR across all three variables were associated with a higher risk stress-induced exhaustion disorder. Physicians who experienced PBR to impact their work *per se*, to impact work negatively or to impact care provision, were approximately twice as likely to report a stress-induced exhaustion disorder. Moreover, physicians who rated low autonomy in patient care and those who reported frequent illegitimate tasks had over four times higher odds of reporting stress-induced exhaustion disorder (OR = 4.33, CI = 4.03–4.66; OR = 4.45, CI = 4.15–4.77). Also, high moral distress is significantly associated with an increased probability of stress-induced exhaustion disorder (OR = 2.85, CI = 2.58–3.16).

**Table 4:** Logistic regression with stress-induced exhaustion disorder as the outcome measure.

	Crude (unadjusted OR)	Model 1	Model 2	Model 3
*n*		828	519	527
Performance-based reimbursement (PBR)				
PBR^a^ impact work (ref. no impact)	2.11 (1.96–2.28)	1.00 (0.88–1.12)		
PBR^a^ impact work negatively (ref. positively)	2.08 (1.83–2.37)		1.58 (1.25–1.99)	
PBR^a^ impact care provision (ref. no impact)	2.11 (1.92–2.32)			1.29 (1.11–1.50)
Other exposures related to stress-induced exhaustion disorder				
Hospitals (ref. primary healthcare facilities)	1.14 (1.05–1.15)	1.45 (1.28–1.64)	1.51 (1.30–1.75)	1.50 (1.29–1.74)
Low autonomy in patient care (ref. high autonomy)	4.33 (4.03–4.66)	2.77 (2.44–3.14)	2.09 (1.79–2.45)	2.19 (1.87–2.55)
High levels of moral distress (ref. low levels)	2.85 (2.58–3.16)	2.12 (1.88–2.39)	2.40 (1.79–2.45)	2.31 (2.00–2.67)
Frequent Illegitimate tasks (ref. seldom)	4.45 (4.15–4.77)	3.78 (3.34–4.27)	3.48 (2.98–4.06)	3.50 (3.00–4.09)
Cox & Snell *R*^2^		0.149	0.150	0.150

Model 1–3, adjusted for work site, autonomy in patient care, moral distress, and illegitimate tasks.

a Performance-based reimbursement.

In Models 1–3 (Table 4), each of the three PBR variables is explored in relation to, autonomy in patient care, moral distress, and frequency of illegitimate tasks, and adjusted for the work site (hospital or primary healthcare facilities). In Model 1, PBR impacted work, becomes non-significant in comparison to crude values. Indicating that the association between PBR and stress-induced exhaustion disorders was moderated by autonomy in patient care, moral distress, and frequency of illegitimate tasks.

In Models 2 and 3, ORs for PBR decreased but remained significant, indicating that PBR was associated with stress-induced stress disorders even when adjusting for the effect of the work site, autonomy in patient care, moral distress, and frequency of illegitimate tasks. In Models 1–3, the OR for autonomy in patient care, moral distress, and frequency of illegitimate tasks decreased compared to crude values, confirming the results from the qualitative analysis. The work site, on the other hand, increased in Models 1–3, indicating that differences existed between physicians working in primary healthcare and hospitals.

To further explore differences between physicians working in primary healthcare and hospitals, a stratified analysis was conducted. Results presented in Table 5 give support that there are important differences between physicians working in primary healthcare and in hospitals. Across crude values, the most evident difference was that in primary healthcare, physicians who experienced PBR to impact their work negatively were five times more likely also to report stress-induced exhaustion disorder (OR = 5.50, CI = 3.47–8.73), whereas the corresponding number for physicians working in hospitals is much lower (OR = 2.07, CI = 1.79–2.40). Across the variables of autonomy in patient care, moral distress, and frequency of illegitimate tasks, ORs were also higher for physicians working in primary healthcare as compared to those working in hospital care.

**Table 5:** Logistic regression with stress-induced exhaustion disorder as the outcome measure stratified for physicians working in primary healthcare (PC) and hospitals (H).

	Crude (unadjusted OR)		Model 1		Model 2		Model 3	
	PC	H	PC	H	PC	H	PC	H
*n*			450	378	332	193	333	194
Performance-based reimbursement (PBR)								
PBR^a^ impact work (ref. no impact)	2.27 (1.96–2.62)	2.25 (2.04–2.48)	0.97 (0.78–1.20)	0.85 (0.72–1.00)				
PBR^a^ impacts work negatively (ref. positively)	5.50 (3.47–8.73)	2.07 (1.79–2.40)			5.38 (2.47–11.71)	1.31 (1.02–1.69)		
PBR^a^ impact care provision (ref. no impact)	2.14 (1.81–2.52)	2.51 (2.17–2.84)					1.02 (0.80–1.31)	1.49 (1.23–1.81)
Other exposures related to stress-induced exhaustion disorder								
Low autonomy in patient care (ref. high autonomy)	5.96 (5.05–7.03)	3.71 (3.39–4.05)	2.97 (2.30–3.83)	2.72 (2.35–3.15)	2.74 (2.04–3.68)	1.91 (1.58–2.31)	2.72 (2.03–3.65)	1.98 (1.64–2.38)
High levels of moral distress (ref. low levels)	3.29 (2.73–3.96)	3.05 (2.67–3.48)	2.27 (1.84–2.80)	2.06 (1.78–2.38)	2.72 (2.16–3.44)	2.16 (1.80–2.59)	2.71 (2.13–3.44)	2.07 (1.72–2.49)
Frequent Illegitimate tasks (ref. seldom)	5.93 (5.09–6.90)	4.21 (3.86–4.58)	3.81 (3.05–4.75)	3.86 (3.33–4.48)	2.79 (2.17–3.59)	3.88 (3.18–4.72)	3.07 (2.38–3.95)	3.84 (3.15–4.68)
Cox & Snell R^2^			0.147	0.150	0.154	0.149	0.144	0.153

Model 1–3, adjusted for work site, autonomy in patient care, moral distress, and illegitimate tasks.

a Performance-based reimbursement scheme.

In Model 1 (Table 5), the ORs for PBR for both primary healthcare and hospital became non-significant. For physicians working in primary healthcare, the ORs for autonomy in patient care and frequency of illegitimate tasks substantially decreased. Such a substantial decrease cannot be identified in the analysis for physicians working in hospitals. This indicates that much of the experiences primary healthcare physicians have in relation to poor autonomy, and frequent illegitimate tasks were linked to PBR. In Model 2, the OR for negative impact of PBR on work remains high in the analysis for primary healthcare, while it decreases in the analysis for hospitals. Thus, the association between PBR impact work negatively, and stress-induced exhaustion disorder was not sensitive to the effect of autonomy in patient care, moral distress, and frequency of illegitimate tasks for physicians in primary healthcare. In Model 3, PBR became non-significant for physicians in primary healthcare, whereas it remained significant for physicians working in hospitals.

## 4. Discussion

In this study, we used the Healthy Healthcare concept to explore how PBR impact on how physicians perceive work, health and patient care, using a mixed-method design. In all, our results indicate that PBR impact Swedish physicians’ perceived work, health and patient care to a large extent and often in a negative way. To the best of our knowledge, no previous study has explored this relationship across a large sample of physicians. The use of a mixed method design gave us the possibility to gain an in-depth understanding of the role PBR might have in physicians’ perceived work, health and patient care. The qualitative analysis showed that physicians experienced money talks, that the care for patients is affected by PBR, that PBR challenges medical morals, and that PBR increases the quantity of illegitimate tasks. The importance of these experiences in relation to physicians’ mental health was further confirmed in the quantitative analysis. Thus, daily financial considerations for the physician in clinical practice seem to disturb crucial aspects of the provision of care. This might have potentially harmful effects on both patients’ care and physicians’ health. Furthermore, the work and health of primary healthcare physicians seem to be affected by PBR to a larger extent, than that of physicians working in hospitals. Moreover, these results further prove, in line with the Healthy Healthcare concept (De Lange et al., 2020), that physicians operate in a complex system and that this system needs to be acknowledged when researching healthcare workers.

Every fifth physician in Sweden and every third primary healthcare physician experienced that PBR impacted their work. Almost all these physicians reported a negative impact of PBR on their work, and that the provision of care was affected. Although lack of autonomy in patient care, moral distress, and the frequency of illegitimate tasks might explain some of the association between PBR and stress-induced exhaustion disorder, PBR also had a direct relationship with stress-induced exhaustion disorder, and this was especially prominent in primary healthcare physicians. Healthcare services globally are currently facing unprecedented challenges, including an ageing population with higher demands for care, general growth in chronic diseases, a need for more personalised medicine, growing health inequalities, and increased public expectations (Liu et al., 2017). To meet these challenges, measures are needed that retain healthcare professionals in their workplace and ensure that resources are optimised (Anand and Bärnighausen, 2012; Bodenheimer and Sinsky, 2014; Sikka et al., 2015; Parkinson, 2018; De Lange et al., 2020). Thus, this study suggests that PBR played an essential role in healthcare provision and that physicians experienced that the reimbursement systems impact their work negatively. The present study shows that there was an association between PBR and stress-induced exhaustion disorders. These results could encourage policymakers to review PBR systems in order to improve physicians’ work and health while meeting future challenges.

One limitation in the study is the cross sectional-design lessening our possibilities to draw causal conclusions. Although additional longitudinal studies are needed, the qualitative analyses strengthen the plausibility that reimbursement systems negatively impact physicians’ work, especially regarding lack of autonomy in patient care, moral distress, and the frequency of illegitimate tasks. Previous longitudinal studies showed that these are risk factors for mental health problems and long-term sickness absence (Elovainio et al., 2013; Albrecht et al., 2017; Åhlin et al., 2018; Williamson et al., 2018; van Hoffen et al., 2021), especially for workers in the public sector such as health care (Elovainio et al., 2013).

Our findings suggest that PBR is associated with physicians experiencing low autonomy in patient care, high moral distress, and an increase in illegitimate tasks, which in turn is associated with stress-induced exhaustion disorder. These links reflect a conflict between how patients ought to be cared for and the lack of resources (Pache and Santos, 2010). Moral distress constitutes a substantial source of mental health morbidity and is a risk factor for stress-induced exhaustion disorder (Glasberg et al., 2007; Førde and Aasland, 2008; Williamson et al., 2018; Kopacz et al., 2019). Similarly, illegitimate tasks impact healthcare professionals’ health negatively (Aronsson et al., 2012; Thun et al., 2018; Kilponen et al., 2021). Results from this study indicate that PBR challenges medical practice and limits physicians’ autonomy to carry out medical management. Physicians also experience that they cannot focus on and give care to those who need it the most. Central practices in physicians’ identity are sound clinical decision-making and medical management, as well as being compassionate, empathic, a good listener, responsive, humane, and honest (O’Donnabhain and Friedman, 2018; Steiner-Hofbauer et al., 2018). Patient-doctor communication is an important factor in modern medicine (Sackett et al., 1996) which, our results suggests, physicians cannot fulfil in a satisfactory manner. In future studies, autonomy in patient care, moral distress, and frequency of illegitimate tasks should be further investigated as a mediator between PBR and stress-induced exhaustion disorder.

Finally, the results of this study confirm the findings of Vengberg et al. (2021) that physicians experience that money talks, that the system supports skimming for healthy patients, and that the most vulnerable and sick patients are being pushed out. It is alarming that many PBR systems seem to be constructed in a way that disadvantages those in most need of care. A well-functioning reimbursement system needs to take all aspects of care and patient work into consideration and adjust for how severely sick patients are.

## 5. Conclusion

Results from this mixed method study suggest that there are strong associations between the current reimbursement systems in Sweden and physicians’ experiences of low autonomy in patient care, high moral distress, and an increase of illegitimate tasks, which in turn is associated with an increased risk of stress-induced exhaustion disorder on the physicians’ behalf. In future research, it is important to investigate whether there are reimbursement systems that are better or worse for healthcare professionals’ work and health. The results indicate that PBRs need to be revised to improve the working conditions for physicians and utmost the care of patients.

## Data availability statement

The data analyzed in this study is subject to the following licenses/restrictions: the sharing of data is restricted due to ethical reasons. Requests to access these datasets should be directed to emma.brulin@ki.se.

## Ethics statement

The Swedish Ethical Review Authority approved this study (2020-06613). Before participation, all respondents were informed about the study and their rights to opt-out.

## Author contributions

EB and AW designed the study. EB took the lead in conducting analyses and writing the manuscript. KE, BL, UL, MS, and AW provided critical feedback and helped shape the research, analysis, and manuscript. All authors contributed to the article and approved the submitted version.

## Funding

This study was funded by Swedish Research Council for Health, working life and Welfare (2019-00311).

## Conflict of interest

The authors declare that the research was conducted in the absence of any commercial or financial relationships that could be construed as a potential conflict of interest.

## Publisher’s note

All claims expressed in this article are solely those of the authors and do not necessarily represent those of their affiliated organizations, or those of the publisher, the editors and the reviewers. Any product that may be evaluated in this article, or claim that may be made by its manufacturer, is not guaranteed or endorsed by the publisher.
